# Supplementary material for: Phytochemical Compounds and Anticancer Activity of Cladanthus mixtus Extracts from Northern Morocco
Source: Cancers (Basel). 2022 Dec 27;15(1):152. doi: 10.3390/cancers15010152 (PMC9818270; doi:10.3390/cancers15010152)
Supplement: Supplementary file 1 [file cancers-15-00152-s001.zip › cancers-2082957-supplementary.pdf]

cancers MDPI

SUPPLEMENTAL INFORMATION

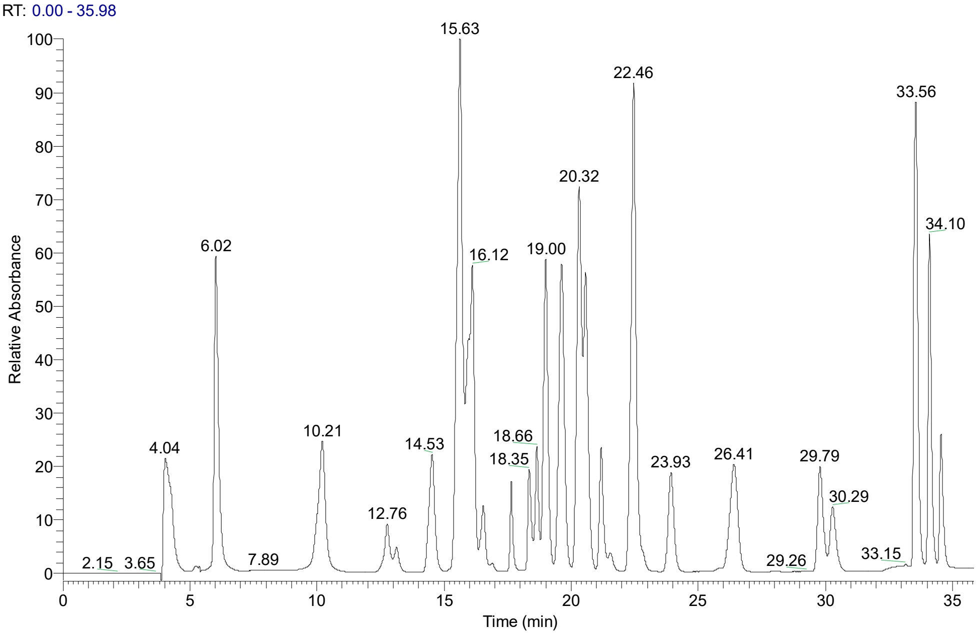

Figure S1. LC–MS chromatogram of standards phenolic compounds in the methanol–water.

Table S1. Standards used to identify and quantify phenolic compounds in *Cladanthus mixtus*.

Peak	Phenolic compound	Retention time (min)	Masse (m/z)	Calibration curve	Coefficient of determination (R2)
1	Gallic acid	6,02	169,43	Y = 52,23*X - 127,5	0,9978
2	Protocatechuic acid	10,21	153,46	Y = 34,24*X - 22,30	0,9994
3	Chlorogenic acid	12,76	353,05	Y = 9,013*X - 21,10	0,9977
4	Catechin	13,13	289,31	Y = 3,962*X + 0,5337	0,9994
5	p-hydroxybenzoïc acid	14,53	137,43	Y = 24,77*X - 9,242	0,9987
6	Caffeic acid	15,63	179,41	Y = 97,26*X + 142,7	0,999
7	Vanillic acid	15,97	167,00	Y = 29,39*X + 16,70	0,9987
8	Syringic acid	16,12	197,00	Y = 44,26*X + 26,40	0,9997
9	Rutin	17,65	609,18	Y = 8,200*X - 1,036	0,999
10	Ellagic acid	18,35	301,22	Y = 12,18*X + 54,35	0,9982
11	Luteolin-7-O-glucoside	18,66	447,00	Y = 14,78*X + 3,791	0,9992
12	p-Coumaric acid	19,00	164,00	Y = 50,04*X - 48,94	0,9986
13	Vanillin	19,63	151,20	Y = 61,85*X - 25,55	0,9984
14	Ferulic acid	20,32	193,00	Y = 65,81*X - 93,12	0,9982
15	Naringin	20,57	579,31	Y = 47,61*X - 38,64	0,9987
16	Apigenin-7-O-glucoside	21,18	431,22	Y = 17,00*X - 3,910	0,9983
17	Rosmarinic acid	22,46	359,10	Y = 87,94*X - 5,717	0,9993
18	Salicylic acid	23,93	137,16	Y = 19,27*X + 1,614	0,9992
19	Methyl paraben	26,41	151,53	Y = 29,78*X + 0,7684	0,9991
20	Luteolin	29,79	285,35	Y = 20,42*X - 15,82	0,9983
21	Quercetin	30,29	301,00	Y = 13,50*X - 5,296	0,999

22	Apigenin	33,56	269.69	Y = 66,91*X + 41,21	0,9993
23	Kaempferol	34,10	285,45	Y = 38,06*X - 21,28	0,9988
24	Isorhamnetin	34,55	315,26	Y = 15,74*X + 19,51	0,9993

A

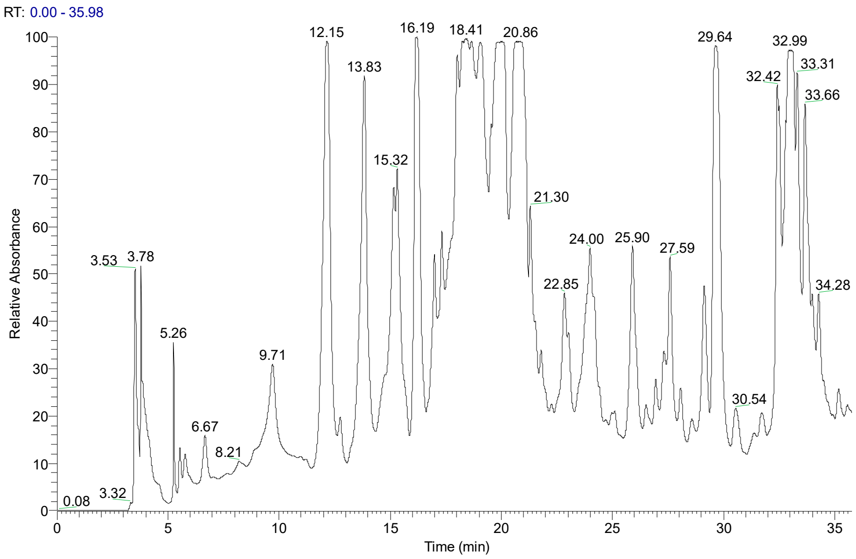

B

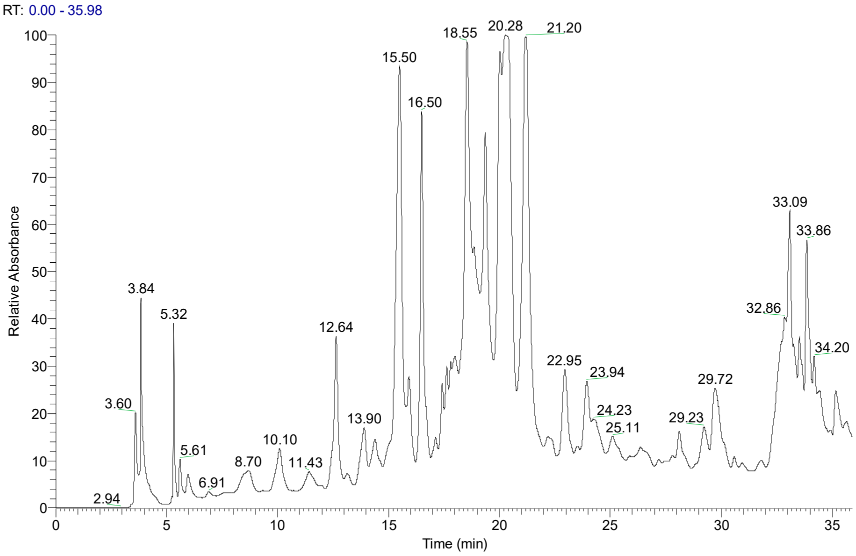

C

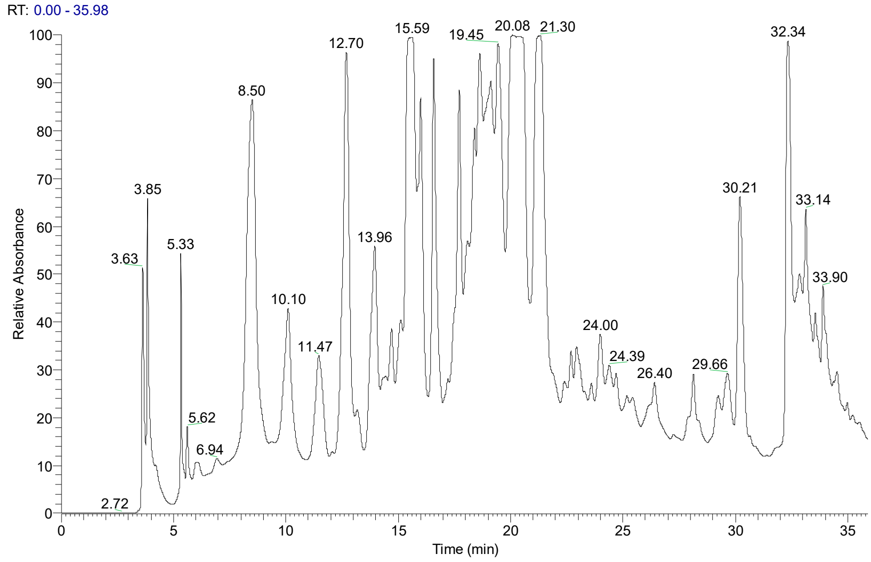

D

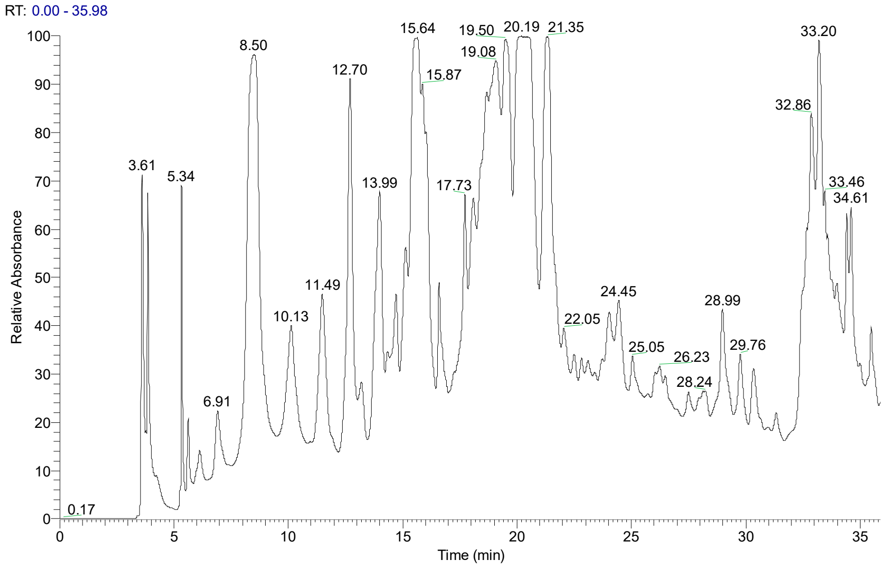

Figure S2. HPLC-MS chromatograms of *Cladanthus mixtus* extracts as detected at 280 nm **(A)** flowers, **(B)** leaves, **(C)** stems, **(D)** roots.

Table S2: Chemical composition of terpenoids obtained by GC-MS.

Molecules	Chemical formula	Molecular weight	Area (%)			
			**Flowers (CM-F)**	**Leaves (CM-L)**	**Stems (CM-S)**	**Roots (CM-R)**
β-sitosterol	$C_{29}H_{50}O$	414.7	6.25	--	--	--
Lupeol	$C_{30}H_{50}O$	426.7	--	14.66	--	8.69
Phytol	$C_{20}H_{40}O$	296.5	--	11.39	6.41	0.97
Eucalyptol	$C_{10}H_{18}O$	154.2	0.48	3.95	2.83	1.22
Campesterol	$C_{28}H_{48}O$	400.7	2.81	1.28	--	--
Taraxasterol	$C_{30}H_{50}O$	426.7	0.87	--	--	--

2,6-Octadienal, 2,6-dimethyl-8-(tetrahydro-2H-2-pyranyloxy)	$C_{15}H_{24}O_3$	252.3	--	6.88	--	--
Neophytadiene	$C_{20}H_{38}$	278.5	--	2.16	--	--
Tetradecane, 2,6,10-trimethyl	$C_{17}H_{36}$	240.5	--	2.38	--	--
Pentadecan-2-one, 6,10,14-trimethyl	$C_{18}H_{36}O$	268.5	--	1.93	0.85	--
Isoshyobunone	$C_{15}H_{24}O$	220.3	--	1.00	--	--
Caryophyllene oxide	$C_{15}H_{24}O$	220.3	--	0.57	--	--
(3α)-D:A-Friedooleanan-3-ol	$C_{30}H_{52}O$	428.7	--	--	1.23	--
**Total**			10.41	46.20	11.32	10.88

(--) : Not detected

Table S3: Chemical composition of esters obtained by GC-MS.

			Area (%)			
**Molecules**	**Chemical formula**	**Molecular weight**	**Flowers (CM-F)**	**Leaves (CM-L)**	**Stems (CM-S)**	**Roots (CM-R)**
Palmitic acid β-monoglyceride	$C_{19}H_{38}O_4$	330.5	7.88	6.32	15.66	13.08
Stearic acid β-monoglyceride	$C_{21}H_{42}O_4$	358.6	3.29	3.21	4.00	5.57
Palmitic acid, methyl ester	$C_{17}H_{34}O_2$	270.5	1.02	--	2.33	1.63
Linoleic acid, methyl ester	$C_{19}H_{34}O_2$	294.5	0.92	--	--	--
Oleic acid, methyl ester	$C_{19}H_{36}O_2$	296.4	0.77	--	--	--
l-Norvaline, N-ethoxycarbonyl-, hexadecyl ester	$C_{24}H_{47}NO_4$	413.6	--	--	3.49	--
l-Proline, N-allyloxycarbonyl-, undec-10-enyl ester	$C_{20}H_{33}NO_4$	351.5	--	--	2.98	--
Diisooctyl phthalate	$C_{24}H_{38}O_4$	390.6	--	--	1.65	--
Artemisyl acetate	$C_{12}H_{20}O_2$	196.3	--	2.47	--	--
Phytyl decanoate	$C_{30}H_{58}O_2$	450.8	--	0.82	--	--
Carbonic acid	$C_3H_4O_3$	88.0	--	0.39	--	--
2-Mercapto-2-methyl-propionic acid	$C_4H_8O_2S$	120.1	--	--	--	1.09
Benzoic acid, 4-butoxy-3-methoxy-perhydro-1-quinolizinyl-methyl ester	$C_{22}H_{33}NO_4$	375.5	--	--	--	0.54
**Total**			13.88	13.21	30.11	21.91

(--) : Not detected

Table S4: Chemical composition of alcohols obtained by GC-MS.

			Area (%)			
**Molecules**	**Chemical formula**	**Molecular weight**	**Flowers (CM-F)**	**Leaves (CM-L)**	**Stems (CM-S)**	**Roots (CM-R)**
Ethyl iso-allocholate	$C_{26}H_{44}O_5$	436.6	--	--	--	23.28
2,3-Dimethyl-3-pentanol	$C_7H_{16}O$	116.2	--	--	--	0.87
1-Hexadecanol	$C_{16}H_{34}O$	242.4	--	--	--	0.34
Diallyl methyl carbinol	$C_8H_{14}O$	126.2	--	5.39	--	--
2-Methyl-*Z*,*Z*-3,13-octadecadienol	$C_{19}H_{36}O$	280.5	--	1.09	--	--

1-Methylcycloheptanol	$C_8H_{16}O$	128.2	--	0.26	--	--
1-Cyclohexanol, 1-[5-hydroxy-4-methyl-2-hexenyl]	$C_{13}H_{24}O_2$	212.3	--	--	3.98	--
5-Azacyclodecanol	$C_9H_{19}NO$	157.2	--	--	1.07	--
**Total**			--	6.74	5.05	24.49

(--) : Not detected

Table S5: Chemical composition of alkanes obtained by GC-MS.

Molecules	Chemical formula	Molecular weight	Area (%)			
			**Flowers (CM-F)**	**Leaves (CM-L)**	**Stems (CM-S)**	**Roots (CM-R)**
Eicosane	$C_{20}H_{42}$	282.5	13.46	2.14	1.97	--
Octadecane, 5,14-dibutyl-	$C_{26}H_{54}$	366.7	0.23	--	--	--
Tetracosane	$C_{24}H_{50}$	338.7	0.47	--	--	--
3-Methylpentacosane	$C_{26}H_{54}$	366.7	0.30	--	--	--
9-Octylhexacosane	$C_{34}H_{70}$	478.9	0.68	--	--	--
Cyclohexane, (1,1-dimethylpropyl)-	$C_{11}H_{22}$	154.2	--	0.49	--	--
1,3,5-Trimethyl-2-octadecylcyclohexane	$C_{27}H_{54}$	378.7	--	--	6.17	2.26
6,6-Dimethyl-10-methylene-1-oxa-spiro[4.5]decane	$C_{12}H_{20}O$	180.2	--	--	5.57	--
10-Methyleicosane	$C_{21}H_{44}$	296.6	--	--	2.24	--
11-Decyltetracosane	$C_{34}H_{70}$	478.9	--	--	2.18	--
Tritetracontane	$C_{43}H_{88}$	605.2	--	--	--	0.88
**Total**			15.14	2.63	18.13	3.14

(--) : Not detected

Table S6: Chemical composition of fatty acid obtained by GC-MS.

Molecules	Chemical formula	Molecular weight	Area (%)			
			**Flowers (CM-F)**	**Leaves (CM-L)**	**Stems (CM-S)**	**Roots (CM-R)**
(*Z*)-18-Octadec-9-enolide	$C_{18}H_{32}O_2$	280.4	12.69	--	--	--
Palmitic acid	$C_{16}H_{32}O_2$	256.4	9.1	11.39	--	--
γ -Stearolactone	$C_{18}H_{34}O_2$	282.5	3.8	--	--	--
(*Z*)-13- Docosenamide	$C_{22}H_{43}NO$	337.6	1.17	--	6.18	8.23
Capric acid	$C_{10}H_{20}O_2$	172.2	1.1	--	--	--
(*E*)-3-methoxy-4-nitro-2-butenoic acid	$C_5H_7NO_5$	161.1	--	0.49	--	--
**Total**			27.86	11.88	6.18	8.23

(--) : Not detected

Table S7: Chemical composition of organic acids obtained by GC-MS.

Molecules	Chemical formula	Molecular weight	Area (%)			
			**Flowers (CM-F)**	**Leaves (CM-L)**	**Stems (CM-S)**	**Roots (CM-R)**
Oxalic acid, dihydrazide	$C_2H_6N_4O_2$	118.1	1.88	0.51	2.74	3.47

2,5-Furandione, 3-(2-decenyl)dihydro-	$C_{14}H_{22}O_3$	238.3	0.64	--	--	--
3-Methylbutanoic acid	$C_5H_{10}O_2$	102.1	--	--	--	1.23
1-Propylheptyl ethylphosphonofluoridate	$C_{12}H_{26}FO_2P$	252.3	--	1.16	--	--
3-Methylcyclopentyl acetate	$C_8H_{14}O_2$	142.2	--	0.18	--	--
**Total**			3.07	1.85	2.74	4.70

(--) : Not detected

Table S8: Chemical composition of benzene and its derivatives obtained by GC-MS.

			**Area (%)**			
**Molecules**	**Chemical formula**	**Molecular weight**	**Flowers (CM-F)**	**Leaves (CM-L)**	**Stems (CM-S)**	**Roots (CM-R)**
1-Nitro-3-(propoxymethyl)benzene	$C_{10}H_{13}NO_3$	195.2	--	--	--	8.15
Loliolide	$C_{11}H_{16}O_3$	196.2	1.64	3.53	3.28	--
Benzaldehyde, 4-methyl-	C_8H_8O	120.1	--	--	2.45	--
ethyl 3,4-dihydro-1H-isochromene-1-carboxylate	$C_{12}H_{14}O_3$	206.2	1.21	--	--	--
(3-Nitrophenyl) methanol, 3-methylbutyl ether	$C_{12}H_{17}NO_3$	223.2	--	2.85	--	--
5,5,8a-Trimethyl-3,5,6,7,8,8a-hexahydro-2H-chromene	$C_{12}H_{20}O$	180.2	--	1.24	--	--
**Total**			2.85	7.62	5.73	8.15

(--) : Not detected

Table S9: Chemical composition of phenols obtained by GC-MS.

			**Area (%)**			
**Molecules**	**Chemical formula**	**Molecular weight**	**Flowers (CM-F)**	**Leaves (CM-L)**	**Stems (CM-S)**	**Roots (CM-R)**
Vanillin	$C_8H_8O_3$	152.1	--	--	--	4.21
2-Methoxy-4-vinylphenol	$C_9H_{10}O_2$	150.1	--	0.52	--	1.28
Phenol, 2,6-dimethoxy-	$C_8H_{10}O_3$	154.1	--	--	--	0.51
6-O-Acetyl-1-[[4-bromophenyl] sulfonyl]-β-D-glucoside	$C_{14}H_{17}BrO_8S$	425.2	--	--	1.61	--
4-Hydroxy-2-methylacetophenone	$C_9H_{10}O_2$	150.1	0.41	--	--	--
**Total**			0.41	0.52	1.61	6.00

(--) : Not detected

Table S10: Chemical composition of ketones obtained by GC-MS.

			**Area (%)**			
**Molecules**	**Chemical formula**	**Molecular weight**	**Flowers (CM-F)**	**Leaves (CM-L)**	**Stems (CM-S)**	**Roots (CM-R)**
Cyclobutanone	C_4H_6O	70.0	--	--	2.89	--
2-Propanone, 1-phenoxy-	$C_9H_{10}O_2$	150.1	--	--	1.58	--
Apocynin	$C_9H_{10}O_3$	166.1	--	--	--	1.03
2',5'-Dimethoxyacetophenone	$C_{10}H_{12}O_3$	180.2	--	--	--	0.64

2(4H)-Benzofuranone, 5,6,7,7a-tetrahydro-4,4,7a-trimethyl-, (R)-	$C_{11}H_{16}O_2$	180.2	--	1.25	--	--
**Total**			--	1.25	4.47	1.67

(--) : Not detected

Table S11: Chemical composition of carbonyls obtained by GC-MS.

Molecules	Chemical formula	Molecular weight	Area (%) Flowers (CM-F)	Leaves (CM-L)	Stems (CM-S)	Roots (CM-R)
5-Ethyl-4-methyl-3-heptanone	$C_{10}H_{20}O$	156.2	19.27	--	--	--
4-Heptanol, 4-ethyl-2,6-dimethyl-	$C_{11}H_{24}O$	172.3	--	1.34	--	--

(--) : Not detected

Table S12: Chemical composition of amines obtained by GC-MS.

Molecules	Chemical formula	Molecular weight	Area (%) Flowers (CM-F)	Leaves (CM-L)	Stems (CM-S)	Roots (CM-R)
N-Butylcyclohexylamine	$C_{10}H_{21}N$	155.2	--	--	6.9	--
9-Octadecenamide, (*Z*)-	$C_{18}H_{35}NO$	281.5	--	1.67	--	--
N-Isobutylideneisobutylamine	$C_8H_{17}N$	127.2	--	0.65	--	--
2(Ethylenedioxy)ethylamine,*N*-methyl-*N*-[4-(1-pyrrolidinyl)-2-butynyl]-	$C_{14}H_{24}N_2O_2$	252.3	--	--	--	0.7
4H-Pyran-4-one, 2,3-dihydro-3,5-dihydroxy-6-methyl-	$C_6H_8O_4$	144.1	1.62	--	--	--
**Total**			1.62	2.32	6.9	0.7

(--) : Not detected

Table S13: Chemical composition of pyrrolidines obtained by GC-MS.

Molecules	Chemical formula	Molecular weight	Area (%) Flowers (CM-F)	Leaves (CM-L)	Stems (CM-S)	Roots (CM-R)
2-Hydroxy-1-(1'-pyrrolidiyl)-1-buten-3-one	$C_8H_{13}NO_2$	155.1	0.56	--	--	0.96
1-Pyrrolidinylacetonitrile	$C_6H_{10}N_2$	110.1	0.18	--	0.74	0.33
1-Propanamine, *N*,*N*,2-trimethyl-2-[(2,2,3-trimethyl-1-pyrrolidinyl)oxy]-, (*S*)-	$C_{13}H_{28}N_2O$	228.3	0.23	--	--	--
1-(1'-pyrrolidinyl)-2-propanone	$C_7H_{13}NO$	127.1	--	--	0.91	--
Pyrrolidine, *N*-(3-methyl-3-butenyl)-	$C_9H_{17}N$	139.2	--	0.22	1.39	--
**Total**			0.97	0.22	3.04	1.29

(--) : Not detected

Table S14: Chemical composition of pyrimidines and steroids obtained by GC-MS.

Molecules	Chemical formula	Molecular weight	Area (%)			
			Flower	Leaf	Stem	Root
**Pyrimidines**						
2(1H)-Pyrimidinethione, tetrahydro-1,3-dimethyl-	$C_6H_{12}N_2S$	144.2	--	--	--	2.07
**Steroids**						
2-Methylene-5alpha-cholestan-3beta-ol	$C_{28}H_{48}O$	400.7	--	2.32	--	--

(--) : Not detected

Table S15: Chemical composition of other molecules obtained by GC-MS.

Molecules	Chemical formula	Molecular weight	Area (%)			
			Flower	Leaf	Stem	Root
5-Piperidin-1-yl-furan-2-carbaldehyde	$C_{10}H_{13}NO_2$	179.2	1.69	--	--	--
5-(4Nitrophenoxymethyl) furan-2-carbaldehyde)	$C_{12}H_9NO_5$	247.2	--	--	--	0.64
2,3-Dihydrobenzofuran	C_8H_8O	120.1	1.46	--	--	--
2-Methylpentadecane-2-thiol	$C_{16}H_{34}S$	258.5	--	0.86	--	--
Triphenylphosphine oxide	$C_{18}H_{15}OP$	278.3	--	--	3.43	--
Docosyl octyl ether	$C_{30}H_{62}O$	438.8	--	0.62	--	--
Butyl(dimethyl)propoxysilane	$C_9H_{22}OSi$	174.3	--	--	1.29	--
Guanine-cytosine	$C_9H_{10}N_8O_2$	262.2	--	--	--	1.28
Hygrine	$C_8H_{15}NO$	141.2	--	--	--	0.38
Cycloheptane, 1,2-dichloro-, cis-	$C_7H_{12}C_{12}$	167.0	--	--	--	1.07
Heptadecane, 9-hexyl-	$C_{23}H_{48}$	324.6	--	--	--	2.79
Normephedrone	$C_{10}H_{13}NO$	163.2	--	--	--	0.61
Vinyl decanoate	$C_{12}H_{22}O_2$	198.3	--	0.4	--	--
**1,5-Heptadiene, 1,5-bis(trimethylsilyl-3-methylene-, 1*E*,5*Z*-**	C_7H_{12}	96.17	1.37	--	--	--
**Total**			4.52	1.88	4.72	6.77

(--) : Not detected.
